# Development and evaluation of a model for predicting the risk of healthcare-associated infections in patients admitted to intensive care units

**DOI:** 10.3389/fpubh.2024.1444176

**Published:** 2024-09-12

**Authors:** Jin Wang, Gan Wang, Yujie Wang, Yun Wang

**Affiliations:** ^1^Department of Healthcare-Associated Infection Management, Qingdao Municipal Hospital, University of Health and Rehabilitation Sciences, Qingdao, China; ^2^Shanghai Institute of Infectious Disease and Biosecurity, Fudan University, Shanghai, China; ^3^School of Public Health, Fudan University, Shanghai, China; ^4^Department of Clinical Laboratory, Qingdao Municipal Hospital, University of Health and Rehabilitation Sciences, Qingdao, China; ^5^Emergency Intensive Care Unit, Qingdao Municipal Hospital, University of Health and Rehabilitation Sciences, Qingdao, China

**Keywords:** machine learning, prediction, risk factors, intensive care, healthcare-associated infections

## Abstract

This retrospective study used 10 machine learning algorithms to predict the risk of healthcare-associated infections (HAIs) in patients admitted to intensive care units (ICUs). A total of 2,517 patients treated in the ICU of a tertiary hospital in China from January 2019 to December 2023 were included, of whom 455 (18.1%) developed an HAI. Data on 32 potential risk factors for infection were considered, of which 18 factors that were statistically significant on single-factor analysis were used to develop a machine learning prediction model using the synthetic minority oversampling technique (SMOTE). The main HAIs were respiratory tract infections (28.7%) and ventilator-associated pneumonia (25.0%), and were predominantly caused by gram-negative bacteria (78.8%). The CatBoost model showed good predictive performance (area under the curve: 0.944, and sensitivity 0.872). The 10 most important predictors of HAIs in this model were the Penetration Aspiration Scale score, Braden score, high total bilirubin level, female, high white blood cell count, Caprini Risk Score, Nutritional Risk Screening 2002 score, low eosinophil count, medium white blood cell count, and the Glasgow Coma Scale score. The CatBoost model accurately predicted the occurrence of HAIs and could be used in clinical practice.

## 1 Introduction

The prevalence of healthcare-associated infections (HAIs) in intensive care units (ICUs) remains high owing to the use of invasive procedures (1), prolonged antibiotic use, and critical patient conditions, having a negative impact on quality of life and healthcare costs (2–5). Early identification and quantification of the risk of HAIs, coupled with timely interventions, are critical for reducing the incidence of HAIs and improving patient outcomes (6). Machine learning (ML) technology has emerged as a useful tool among healthcare professionals for predicting prognosis and making treatment decisions. Use of ML applications in ICU research has shown a 22.2% improvement in predicting complications (7). Although existing research often relies on hospitalization data for infection prediction, early initiation of infection control measures is important to improving patient outcomes (8–10). This study aimed to use indicators collected within 24 h of admission to the ICU using ML technology to develop infection prediction models with an earlier predictive window. Notably, the enhanced predictability of ML models, albeit at the expense of interpretability, makes it a reliable tool for predicting specific medical conditions.

Previous studies have used ML techniques to refine risk prediction in the healthcare context. Within ICUs, the Simplified Acute Physiology Score (SAPS) II has shown moderate accuracy for predicting HAIs and 7-day mortality (11). However, combining SAPS II with additional patient characteristics using support vector machine algorithms increased the predictive accuracy for both outcomes (12). In the neonatal ICU, a generalized mixed-effects regression tree with random intercept (GMERT-RI) model identified key predictors of HAIs, highlighting the importance of healthcare-associated factors in addition to neonate-specific factors (13). These findings highlight the potential of ML models to refine risk prognostication in critical care environments and facilitate targeted interventions to improve patient outcomes.

Early identification of HAI risk, enables early intervention; however, relatively few studies have focused on predicting HAIs using data collected within 24 h of ICU admission. This retrospective study used data from patients admitted to the ICU, focusing on the clinical characteristics of patients who developed infections during hospitalization. Multiple ML techniques were used to develop infection prediction models, leading to the development of a comprehensive model for training. These models identified significant predictors and key determinants which can be used the help healthcare professionals in the early identification of infection risk among patients admitted to ICUs, facilitating timely intervention.

## 2 Methods

### 2.1 Study participants

The study participants were patients admitted to the ICU of a tertiary grade A comprehensive hospital in China between January 2019 and December 2023, with a total of 3,173 patients. We retrospectively analyzed the patients’ demographic characteristics, blood test results, nursing scores, and invasive clinical procedures within 24 h of admission. The data on patient demographics and invasive clinical procedures were extracted from the infection information monitoring system of Xinglin Hospital, blood markers were extracted from the Ruimei laboratory management system, and nursing scores were extracted from the intensive care information system.

The inclusion criteria were: 1. ICU admissions between January 2019 and December 2023; 2. Hospital stays exceeding 48 h; 3. The data within 24 h of ICU admission; 4. Infection-related data including occurrence, site, and pathogens throughout the hospitalization period. According to the “HAIs Diagnosis Criteria (Trial)” (14) in China, HAIs that occur in the hospital without a clear incubation period are classified as HAIs if they manifest 48 h or more after admission. Consequently, the 48-h timeframe serves as a critical criterion for identifying hospital infections. This study focused on patients who were admitted to the ICU for more than 48 h, in adherence to this guideline.

The exclusion criteria were: 1. Exclusion of colonization, community-acquired infections, and contamination in infection diagnoses (*N* = 21); 2. Exclusion of patients with disputed infection diagnoses (*N* = 5); 3. Exclusion of patients not discharged by December 31, 2023 (*N* = 25); 4. Data incompleteness within 24 h of ICU admission exceeds 5.0% (*N* = 23). Infection diagnosis adhered to the “HAIs Diagnosis Criteria (Trial)” (14) standard, considering the detection of the same pathogen at the same site in the same patient during hospitalization as one infection.

Following screening, the study encompassed a total of 2,517 cases, including 455 instances of HAIs.

### 2.2 Data collection

The data included 32 factors though to be relevant to predicting the infection risk among ICU patients. The patient characteristics included gender, age, and diabetes status. Blood test results included white blood cell (WBC) count, neutrophil count, monocyte count, lymphocyte count, eosinophil count, basophil count, red blood cell (RBC) count, hemoglobin level, RBC distribution width, hematocrit, mean corpuscular hemoglobin, mean corpuscular hemoglobin concentration, mean corpuscular volume, platelet count, C-reactive protein level, glucose level, albumin level, and total bilirubin level. The nursing scores consisted of the Braden score, Nutritional Risk Screening (NRS) 2002 score, Glasgow Coma Scale (GCS) score, Critical Care Pain Observation Tool (CPOT), Penetration Aspiration Scale (PAS), enteral feeding tolerance, Caprini Risk Score (CRS), and unplanned extubation assessment (2, 15, 16). Invasive procedures included mechanical ventilation, intravascular catheterization, and urinary catheterization. The value of each indicator was determined based on the first test or assessment conducted within 24 h of ICU admission. The primary outcome was the occurrence of HAIs among patients admitted to the ICU.

### 2.3 Ethical approval

To respect and protect the legitimate rights and interests of the subjects, this research has been approved by the Ethics Committee of Qingdao Municipal Hospital (approval no: 2024-KY-020). The individual patient consent requirement is waived because the project does not affect clinical treatment of patients, poses no greater than minimal risk, and all protected health-sensitive information has been removed from the limited dataset used in this study.

### 2.4 Identification of pathogens on culture

In compliance with the specifications outlined in the WS/T 640—2018 Clinical Microbiology Examination: Sample Collection and Transportation (17), clinical specimens were collected for culture from patients with suspected HAIs. Bacteria were identified using fully automated microbial identification and drug sensitivity analysis systems such as VITEK 2 (bioMérieux, Marcy-L’Étoile, France) or matrix-assisted laser desorption ionization time-of-flight mass spectrometry (MALDI-TOF MS; Bruker Daltonics, GmbH, Bremen, Germany). In patients with more than one bacterial strain isolated from various specimen types during their ICU admission, only the first pathogen identified was included in the analysis.

### 2.5 Statistical analysis

During the data processing phase, the researchers performed data anonymization to ensure that evaluators could not access background information related to the sample results. The data for Neutrophil Count, glucose levels, albumin levels, total bilirubin levels, and C-Reactive Protein have some missing values; however, the missing data for each is below 5.0%. The researchers filled in these missing values using the mean of the respective columns. This study used chi-square tests and t-tests to assess the statistical significance of differences in categorical and continuous variables, respectively, to identify predictors of infection in patients with and without HAIs (18, 19), to mitigate the risk of overfitting and enhance the interpretability of the model. As hypothesis testing using chi-square tests and t-tests only assessed the statistical significance of differences between groups, and did not necessarily identify the best predictors, variable selection algorithms and cross-validation techniques were used for variable selection (20). Multiple methodologies were used to construct a prediction model that was both accurate and robust.

### 2.6 Model building and evaluation

Data processing and analysis were performed using Python pandas, and Python Scikit-learn was used for model construction. OneHotEncoder was used to transform categorical variables into one-hot encoding (21), and StandardScaler was used to standardize numerical variables and enhance model performance and convergence speed (22). This study used ColumnTransformer to combine two distinct variable-processing methods in ML. The dataset is divided into training and testing sets with an 80–20 split. This means 80.0% of the data is used for training the model, and 20.0% is reserved for testing the model. This study used 10 commonly used machine learning models: Random Forest (23), eXtreme Gradient Boosting (XGBoost) (24), Multilayer Perceptron (MLP), Adaptive Boosting (AdaBoost), Logistic Regression (25), Light Gradient Boosting Machine (LightGBM) (26), Decision Tree (27), Naive Bayes, Neural Networks (28), and Categorical Boosting (CatBoost) (29). The least absolute shrinkage and selection operator (LASSO) was used for variable selection (23). The predictive performance of each model was assessed using the area under the receiver operating characteristic (ROC) curve (AUC), sensitivity, specificity, and Youden’s index (30, 31). In this study, the optimal thresholds for the best model were determined using Youden’s J statistic in the test dataset. Additionally, all model output results were generated by an automated system, which further minimized the potential for human intervention, thereby enhancing the reliability of the evaluation results. The code has been shared on GitHub, please visit https://github.com/aarontroy/HAIs-prediction-model.git.

## 3 Results

### 3.1 Patient characteristics

A total of 2,517 patients, including 1,544 male and 973 female patients with a mean age of 66.191 ± 19.067 years, met the eligibility criteria and were included in the analysis. Of the patients, 706 had been diagnosed with diabetes. In this cohort, 455 patients (18.1%, 309 male and 146 female patients; mean age 70.160 ± 18.403 years) developed HAIs (Table 1). Comparison of the characteristics of patients with and without HAIs using chi-square tests and t-tests, identified 18 statistically significant variables including gender, WBC count, RBC count, lymphocyte count, hemoglobin level, total bilirubin level, RBC distribution width, hematocrit, mean corpuscular hemoglobin concentration, mean corpuscular volume, eosinophil count, age, Braden score, NRS 2002 score, GCS score, PAS, enteral feeding tolerance, and the CRS.

**Table 1:** Baseline characteristics of patients.

Dependent variable	Group	HAIs	Non-HAIs	Chi-square value/*t*-test	*p*-value
Gender	Male	309 (67.9)^a^	1,235 (59.9)	9.772	0.002
	Female	146 (32.1)	827 (40.1)		
Diabetes status	Yes	117 (25.7)	589 (28.6)	1.363	0.240
	No	338 (74.3)	1,473 (71.4)		
WBC count (10^9/L)	<3.5	18 (4.0)	92 (4.5)	8.514	0.014
	3.5–9.5	194 (42.6)	729 (35.3)		
	>9.5	243 (53.4)	1,241 (60.2)		
Neutrophil count (10^9/L)	<1.8	38 (8.3)	192 (9.3)	2.392	0.302
	1.8–6.3	161 (35.4)	654 (31.7)		
	>6.3	256 (56.3)	1,216 (59.0)		
Monocyte count (10^9/L)	<0.1	16 (3.5)	88 (4.3)	0.855	0.652
	0.1–0.6	264 (58.0)	1,158 (56.2)		
	>0.6	175 (38.5)	816 (39.5)		
Lymphocyte Count (10^9/L)	<1.1	266 (58.5)	1,345 (65.2)	7.559	0.023
	1.1–3.2	179 (39.3)	674 (32.7)		
	>3.2	10 (2.2)	43 (2.1)		
Eosinophil count (10^9/L)	0–0.06	444 (97.6)	1991 (96.6)	0.940	0.332
	>0.06	11 (2.4)	71 (3.4)		
Basophil count (10^9/L)	<0.02	175 (38.4)	1,080 (52.4)	36.689	<0.001
	0.02–0.52	256 (56.3)	937 (45.4)		
	>0.52	24 (5.3)	45 (2.2)		
RBC Count (10^12/L)	Male<4.3/Female<3.8	331 (72.7)	1,322 (64.1)	13.725	0.001
	Male4.3–5.8/Female3.8–5.1	117 (25.7)	672 (32.6)		
	Male>5.8/Female>5.1	7 (1.6)	68 (3.3)		
Hemoglobin level (g/L)	Male<130/Female<115	332 (72.9)	1,362 (66.1)	8.524	0.014
	Male130-175/Female115-150	114 (25.1)	635 (30.8)		
	Male>175/Female>150	9 (2.0)	65 (3.1)		
RBC distribution width (fl)	<37	7 (1.5)	20 (1.0)	29.190	<0.001
	37–54	323 (71.0)	1,694 (82.1)		
	>54	125 (27.5)	348 (16.9)		
Hematocrit (%)	Male<40/Female<35	340 (74.7)	1,426 (69.2)	6.167	0.046
	Male40-50/Female35-45	102 (22.4)	545 (26.4)		
	Male>50/Female>45	13 (2.9)	91 (4.4)		
Mean corpuscular hemoglobin (pg)	<27	56 (12.3)	235 (11.4)	0.220	0.639
	27–34	399 (87.7)	1827 (88.6)		
	>34	0 (0.0)	0 (0.0)		
Mean corpuscular hemoglobin concentration (g/L)	<316	139 (30.5)	545 (26.4)	6.374	0.041
	316–354	302 (66.4)	1,407 (68.2)		
	>354	14 (3.1)	110 (5.4)		
Mean corpuscular volume (fl)	<82	19 (4.2)	149 (7.2)	15.733	<0.001
	82–100	368 (80.9)	1716 (83.2)		
	>100	68 (14.9)	197 (9.6)		
Platelet count (10^9/L)	<125	97 (21.3)	491 (23.8)	1.376	0.503
	125–350	323 (71.0)	1,425 (69.1)		
	>350	35 (7.7)	146 (7.1)		
Glucose level (mmol/L)	<3.9	9 (2.0)	71 (3.4)	3.062	0.216
	3.9–6.1	104 (22.8)	435 (21.1)		
	>6.1	342 (75.2)	1,556 (75.5)		
Albumin level (g/L)	<40	440 (96.7)	1995 (96.8)	0.000	1.000
	40–55	15 (3.3)	67 (3.2)		
	>55	0 (0.0)	0 (0.0)		
Total bilirubin level (umol/L)	<3.4	5 (1.1)	11 (0.5)	21.690	<0.001
	3.4–17.1	329 (72.3)	1,271 (61.7)		
	>17.1	121 (26.6)	780 (37.8)		
C-reactive protein (mg/L)	0–6	60 (13.2)	324 (15.7)	1.650	0.199
	>6	395 (86.8)	1738 (84.3)		
Mechanical ventilation	Yes	273 (60.0)	903 (43.8)	38.687	4.975
	No	182 (40.0)	1,159 (56.2)		
Intravascular catheterization	Yes	173 (38.0)	757 (36.7)	0.221	0.638
	No	282 (62.0)	1,305 (63.3)		
Urinary catheterization	Yes	358 (78.7)	1,559 (75.6)	1.776	0.183
	No	97 (21.3)	503 (24.4)		
Age (years)	–	73 (60-86)^b^	68 (54–81)	4.927	<0.001
Braden score	–	10 (10–11)	11 (10–12)	−8.218	<0.001
NRS 2002 score	–	4 (3–5)	4 (3–4)	4.869	<0.001
GCS score	–	3 (3–4)	3 (3–4)	3.793	<0.001
CPOT score	–	1 (0–1)	1 (0–1)	0.348	0.728
PAS	–	8 (6–11)	6 (3–9)	9.034	<0.001
Enteral feeding tolerance	–	1 (0–1)	0 (0–1)	4.361	<0.001
CRS	–	8 (6–10)	7 (5–9)	7.142	<0.001
Unplanned extubation assessment	–	6 (4–8)	6 (4–8)	−0.526	0.599

a The description of continuous variables is expressed as *N* (%).

b Descriptive statistics for categorical variables are presented using median (interquartile range).

### 3.2 Characteristics of healthcare-associated infections

#### 3.2.1 Infection sites

Of the patients included in the analysis, 585 HAIs were identified in 455 patients, including 91 patients who experienced multiple episodes of HAI. HAIs were identified at 14 different sites. The top 5 infected sites were lower respiratory tract (28.7%, 168 cases), ventilator-associated pneumonia (25.0%, 146 cases), Bacteremia (19.3%, 113 cases), Catheter-associated urinary tract infection (9.6%, 56 cases) and Central line-associated bloodstream infection (5.5%, 32 cases) (Table 2).

**Table 2:** Distribution of infected sites in patients in the hospital.

Infection site	Quantity (cases)	Composition ratio (%)
Lower respiratory tract infection	168	28.7%
Ventilator-associated pneumonia	146	25.0%
Bacteremia	113	19.3%
Catheter-associated urinary tract infection	56	9.6%
Central line-associated bloodstream infection	32	5.5%
Urinary tract infection	21	3.5%
Intra-abdominal (pelvic) tissue infection	20	3.4%
Skin and soft tissue infection	9	1.5%
Surgical site infection	5	0.9%
Pleural cavity infection	5	0.9%
Burn infection	4	0.7%
Gastrointestinal infection	3	0.5%
Central nervous system infection	2	0.3%
Arterial catheter-related bloodstream infection	1	0.2%
Total	585	100.0%

#### 3.2.2 Bacterial pathogens identified

Among the 455 patients with HAIs, 396 bacterial pathogens were grown on culture, an 87.0% detection rate. A total of 640 strains were identified, comprising 504 (78.8%) gram-negative bacteria, 94 (14.7%) gram-positive bacteria, and 42 (6.6%) fungi. Of the patients, 217 had a single pathogen identified, and 179 were infected with multiple pathogens. The top five pathogens identified were *Acinetobacter baumannii* (25.5%, 163 cases), *Klebsiella pneumoniae* (17.0%, 109 cases), *Pseudomonas aeruginosa* (14.1%, 90 cases), *Enterobacter cloacae* (5.2%, 33 cases), and *Escherichia coli* (4.8%, 31 cases) (Table 3).

**Table 3:** Distribution of pathogenic microorganisms in infected patients.

Pathogen	Strain (cases)	Composition Ratio (%)
*Acinetobacter baumannii*	163	25.5%
*Klebsiella pneumoniae*	109	17.0%
*Pseudomonas aeruginosa*	90	14.1%
*Proteus mirabilis*	33	5.2%
*Escherichia coli*	31	4.8%
*Staphylococcus aureus*	22	3.4%
*Enterococcus faecalis*	20	3.1%
*Enterobacter cloacae*	17	2.6%
*Brevibacterium* brevis	14	2.2%
*Burkholderia cepacia*	13	2.0%
*Staphylococcus aureus* hemolyticus	11	1.7%
*Acinetobacter lwoffii*	11	1.7%
*Candida albicans*	11	1.7%
*Candida glabrata*	11	1.7%
*Serratia marcescens*	11	1.7%
*Staphylococcus epidermidis*	10	1.6%
*Enterococcus faecium*	9	1.4%
*Bacillus cereus*	9	1.4%
*Candida tropicalis*	8	1.3%
*Staphylococcus hominis*	8	1.3%
*Klebsiella oxytoca*	7	1.1%
*Morganella morganii*	4	0.6%
*Staphylococcus capitis*	4	0.6%
*Aspergillus flavus*	4	0.6%
*Klebsiella oxytoca*	4	0.6%
*Aspergillus fumigatus*	3	0.5%
*Streptococcus pneumoniae*	1	0.2%
*Flavobacterium meningosepticum*	1	0.2%
*Corynebacterium amycolatum*	1	0.2%
Total	640	100.0%

### 3.3 Synthetic minority oversampling technique

The synthetic minority oversampling technique (SMOTE) algorithm was used to inflate the number of patients with HAIs from 445 to 2062, to align the size of the HAI cohort with that of the non- HAI cohort, thereby effectively mitigating class imbalance. This study categorized the model predictions into two groups based on whether oversampling was conducted, thus facilitating a comparative evaluation of the predictive performance.

### 3.4 Variable selection and dimensionality reduction

The LASSO algorithm is a classic linear regression technique that is widely used for variable selection and dimensionality reduction. Compared with traditional linear regression methods, LASSO is capable of automatically selecting variables that have a marked effect on the target variable while preserving predictive accuracy (23). Consequently, this leads to a reduction in model complexity and an enhancement in generalization performance. In the analysis without SMOTE oversampling, the LASSO algorithm excluded the following variables: C-reactive protein, mean corpuscular hemoglobin concentration, GCS score, CPOT score, gastrointestinal tolerance to nutrition, and CRS. Conversely, in the analysis with SMOTE oversampling, the LASSO algorithm excluded gender, CRS, unplanned extubation assessment, CPOT score, diabetes, aspiration/asphyxia score, GCS score, and gastrointestinal tolerance to nutrition.

**Figure 1:**
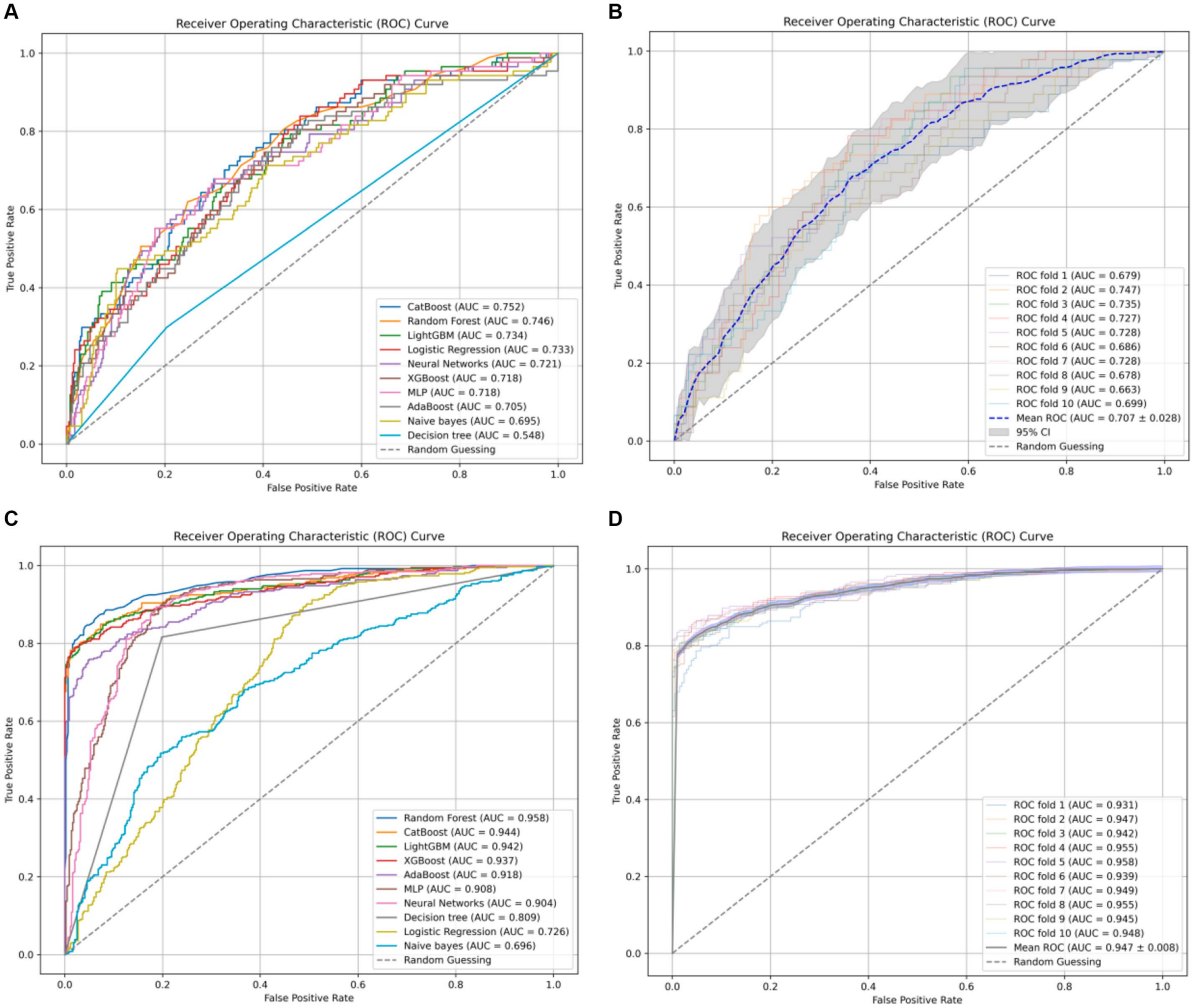
**(A)** The receiver-operating characteristic curves for ten ML models without undergoing SMOTE oversampling processing. **(B)** The ten-fold cross-validation results of the CatBoost model without SMOTE oversampling. **(C)** The receiver-operating characteristic curves for ten ML models with undergoing SMOTE oversampling processing. **(D)** The ten-fold cross-validation results of the CatBoost model with SMOTE oversampling.

Typically, AUC is used as the primary metric for evaluating model performance owing to its comprehensive consideration of both true-positive and false-positive rates (Figure 1). According to the data provided, the Random Forest model demonstrated the best performance in terms of the AUC (0.958). However, given the 18.1% incidence of infection in the study hospital, sensitivity was considered the most important metric of model performance. Notably, the CatBoost model outperformed the others in terms of sensitivity (0.872) at detecting HAIs. In this context, the CatBoost model was considered the best choice (Table 4).

**Table 4:** Evaluation metrics computation of machine learning models without or with undergoing SMOTE oversampling.

Model	AUC		Sensitivity		Specificity		Youden’s J	
	NO_SMOTE	SMOTE	NO_SMOTE	SMOTE	NO_SMOTE	SMOTE	NO_SMOTE	SMOTE
CatBoost	0.752	0.944	0.558	0.872	0.704	0.737	0.262	0.609
Random forest	0.746	0.958	0.547	0.784	0.669	0.772	0.217	0.556
LightGBM	0.734	0.942	0.587	0.842	0.664	0.753	0.251	0.595
Logistic regression	0.733	0.726	0.583	0.564	0.673	0.675	0.256	0.238
Neural networks	0.721	0.904	0.540	0.707	0.688	0.795	0.227	0.501
XGBoost	0.718	0.937	0.558	0.833	0.670	0.724	0.228	0.557
MLP	0.718	0.908	0.522	0.721	0.699	0.795	0.220	0.515
AdaBoost	0.705	0.918	0.568	0.756	0.658	0.735	0.226	0.491
Naive Bayes	0.695	0.696	0.575	0.610	0.626	0.600	0.201	0.210
Decision tree	0.548	0.809	0.433	0.657	0.599	0.651	0.032	0.308

We conducted ten-fold cross-validation using the CatBoost classifier, training the model using preprocessed data (X_processed). In each fold of cross-validation, data splitting was performed using the StratifiedKFold method with 10 folds (n_splits = 10), ensuring data shuffling (shuffle = True) and maintaining consistency by setting a random seed (random_state = 42). Subsequently, the model was fitted to each training set (train) and the probability values were predicted using the corresponding test set (test). The mean AUC was 0.947 ± 0.008. These results highlight the excellent predictive performance and stability of the CatBoost model after SMOTE oversampling. In this study, the optimal thresholds for the CatBoost model were determined using Youden’s J statistic in the test dataset. For the NO_SMOTE group, the optimal threshold was calculated to be 0.558. This threshold allows the model to achieve the best balance between sensitivity and specificity. In the SMOTE group, which addresses class imbalance issues, the optimal threshold was found to be 0.581. This higher threshold helps to further enhance the model’s performance on imbalanced datasets.

**Figure 2:**
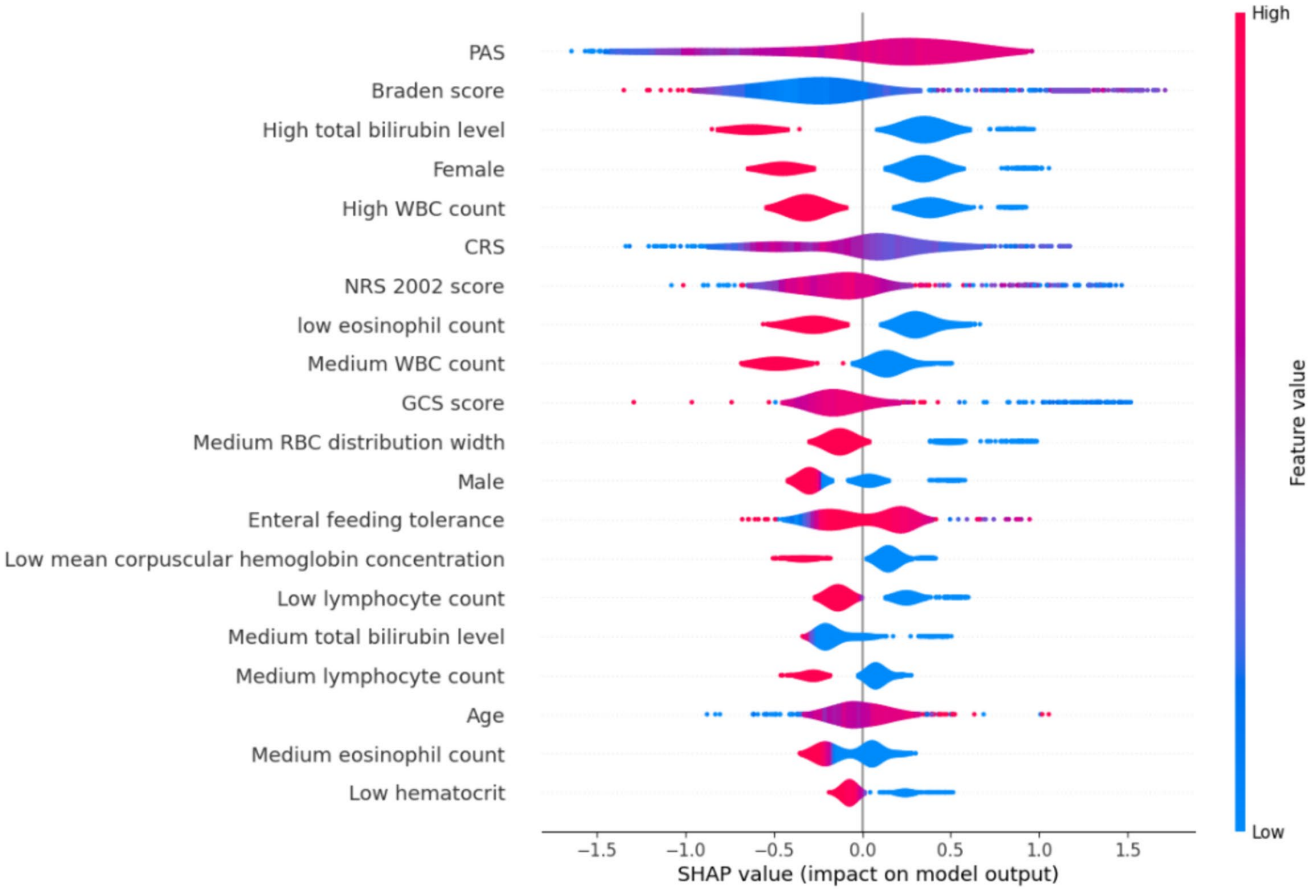
SHapley Additive exPlanation summary plots.

A model was developed to identify factors contributing to HAIs in ICU patients using the CatBoost method. Eighteen independent variables identified through single-factor analysis were used as inputs, and the occurrence of HAIs events was the dependent (outcome) variable. The SHapley Additive exPlanation (SHAP) values were used as a technique to elucidate model predictions, to assist with understanding of individual variable contributions to model predictions. The SHAP values of each selected variable was visualized using violin plots, to reveal the distribution of the impact of variable on the model output. The width of the violin shape denotes the distribution of the SHAP values for the variable; a broader shape signifies a wider range of effects and potentially more diverse effects of the selected predictor variables. In the CatBoost model, the top 10 predictors of HAIs and their SHAP values were PAS (0.470), Braden score (0.470), high total bilirubin level (0.465), female (0.414), high WBC count (0.387), CRS (0.345), NRS 2002 score (0.321), low eosinophil count (0.318), medium WBC count (0.284), and GCS score (0.269) (Figure 2).

## 4 Discussion

This study devised an algorithmic model for predicting the occurrence of HAIs in patients admitted to the ICU. CatBoost was used as the ML model and trained on the full dataset containing 32 variables and a dataset containing 18 selected variables. Subsequent enhancement of model performance was achieved by preprocessing the data using SMOTE oversampling. By using the SHAP method to interpret the findings, this study attained interpretability and transparency in the HAI outcomes predicted using ML at both the population and individual level. The use of ML to predict the occurrence of HAIs has potential application for early alerts and preemptive strategies, facilitating personalized treatment regimens and resource distribution, streamlining the clinical decision-making process and management protocols, and promoting advancements in the quality and safety of medical care.

Machine learning has gradually been applied to clinical sample analysis to handle variable data related to patient infection status (6). AI and ML technologies are expected to develop hospital infection monitoring algorithms to identify risk factors, improve patient risk stratification, track transmission pathways, and achieve real-time infection detection. Electronic health data plays a critical role in this process and is increasingly accessible. Advanced data management systems support real-time decision-making, aiding in automated hospital infection monitoring (32, 33). In the ICU, ML-supported clinical decision research focuses on monitoring, diagnosing, early identification of clinical events, outcome prediction, and prognosis assessment, to assist doctors, researchers, and policymakers in making treatment decisions. Currently, numerous ML models are used to predict risks of VAP, CLABSI, SSI, and hospital-acquired MDR pathogens, with particular emphasis on predicting sepsis and septic shock (6). In hospital infection risk prediction, clinical oversight is crucial to prevent applying machine learning systems to new data that differs from the training data (34), thus minimizing discrepancies between prediction results and actual clinical assessments (35).

The incidence rate of HAIs in ICUs is generally high, which may lead to a prolonged hospital stay and increased disease burden for patients. In this study, the incidence of infection in the ICU was 18.1%. Other studies have reported an incidence of HAIs in ICUs ranging from 8.0 to 60.0% (2, 36). The incidence of HAIs related to invasive procedures ranges from 25.0 to 50.0%, with patients in the ICU having a risk of HAIs 5 to 10 times higher than that in patients in general wards (37). Urinary tract infections related to catheterization in the ICU can increase the mortality rate by approximately 10.0% and prolong the length of hospital stay by an average of 10 days (38). Among the pathogens detected in this study, gram-negative bacteria were predominant, accounting for 78.8% of the cases. This finding is slightly higher than the 75.3% reported by Wang et al. (2) and substantially higher than the 67.4% reported by Li et al. (3) and the 57.4% reported by Cabrera-Tejada et al. (39). HAIs present a significant challenge in the management of ICU patients, often resulting in serious complications. HAIs can significantly affect patient outcomes, leading to increased complexity of treatment, prolonged hospital stays, an increased risk of bacterial resistance, increased mortality rates, an increased risk of disability, and higher healthcare costs (4, 6). In a study conducted by Cabrera-Tejada et al. (4), of patients admitted to the ICU and undergoing invasive mechanical ventilation for over 48 h, the mean length of hospital stay increased by 13.6 days, with an additional treatment cost of 20,965 Euros in patients with HAIs. These findings highlight the substantial burden HAIs pose on healthcare systems and patient outcomes, and the importance of effective prevention and management strategies in ICU setting (4).

Refining unbalanced datasets can enhance the accuracy of classification models used in scientific research. Cleaning unbalanced datasets can enhance the accuracy of classification models (40). The study revealed that compared with random under-sampling and oversampling techniques, predictions using the clustered under-sampling method led to more precise predictions of mortality rates (40). There are three common methods for addressing imbalanced ML data: data-level, algorithm-level, and hybrid approaches (41). In data-level methods, researchers modify the training dataset to suit the classifier algorithms, such as oversampling or under-sampling. Algorithm-level methods involve adjusting existing learners to reduce the bias toward the majority of the population, such as using cost-sensitive methods (41). This study aimed to predict HAIs by applying various balancing methods to the baseline data of ICU patients collected within 24 h of admission. In medical datasets, the records in the minority class are often more crucial than those in the control class. Therefore, addressing imbalanced data is essential to enhance the HAI identification rate. In addition to using SMOTE for oversampling, this study incorporated the LASSO operator for variable selection to reduce model complexity and enhance generalization capabilities.

The optimal model for predicting HAIs risk varies depending on the research context. Zhang et al. (18) found that the Naive Bayes model performed best in predicting surgical site infections after spinal surgery, with a mean AUC of 0.95, a sensitivity of 0.78, a specificity of 0.88, and an accuracy of 0.87. Cho et al. (42) have developed an ML model to monitor site infections during colon surgery. Their study revealed that neural networks using recursive variable elimination with 29 variables showed the best performance, achieving an AUC of 0.963. Previous studies have identified age, hemoglobin level, WBC count, and neutrophil count as predictors of catheter-related bloodstream infections using the XGBoost model (6), consistent with the findings of this study.

Owing to the increased risk of aspiration in ICU patients, inhalation of oropharyngeal secretions or vomitus can easily contaminate respiratory tract hygiene, leading to an increased risk of lower respiratory tract infections (43). Therefore, in patients with high PAS scores, the head of the bed should be elevated by 30° to 45° to reduce the risk of aspiration (43). In this study, the Braden score was identified as another predictor of HAI occurrence. Ding et al. (44) found that a low Braden score was an independent risk factor for stroke-associated pneumonia (SAP) after spontaneous intracerebral hemorrhage, showing moderate effectiveness in predicting SAP. In a study of chemotherapy-related bacterial infections, Jin et al. (45) found that an NRS 2002 score ≥ 3 was an independent risk factor; therefore, improving nutritional status could reduce the occurrence of bacterial infections. The findings of the studies by Ding et al. and Jin et al. were similar to the results of this study. It is crucial to monitor the scores of patients within 24 h of ICU admission and to intervene effectively if indicated.

This study has some limitations. The data were from a single center; therefore, the generalizability of the results may be limited. Multicenter studies should be conducted to enable a more thorough examination of variations according to region, demographics, and clinical practice. The research subjects for hospital infection monitoring using machine learning methods can be tens of thousands of people, which makes it more convenient for training and testing AI algorithms (46). Moreover, using the predictive model in real-world clinical settings, accompanied by real-time performance monitoring, is essential. Furthermore, strategies need to be devised for seamlessly integrating the model into existing medical information systems to facilitate easy access to and use of the predicted outcomes by clinicians. Further research could include additional variables associated with HAIs, such as demographic information (e.g., height and weight), vital sign (e.g., pulse, respiration, blood pressure, and temperature), and laboratory measurements (e.g., arterial blood gases) (47). The only pre-existing medical condition considered in this study was a history of diabetes. Future research should explore additional pre-existing medical conditions as potential predictors of HAIs.

## 5 Conclusion

This study presents the development and analysis of an ML algorithm aimed at predicting the risk of HAIs using data on patients admitted to an ICU collected within 24 h of admission, as predictors. Through a meticulous examination of the ranking of predictors derived from the ML model, this study identified key risk factors associated with HAIs, facilitating the identification of at-risk patients and the formulation of personalized treatment strategies. Future studies should include additional potential predictor variables, multicenter data, and a larger sample size to enhance the accuracy of prediction outcomes.

## Data Availability

The raw data supporting the conclusions of this article will be made available by the authors, without undue reservation.

## References

[1] Migliara G Di Paolo C Barbato D Baccolini V Salerno C Nardi A. Multimodal surveillance of healthcare associated infections in an intensive care unit of a large teaching hospital. Ann Ig. (2019) 31:399–413. doi: 10.7416/ai.2019.2302, PMID: 31304521

[2] Wang Y Ren J Yao Z Wang W Wang S Duan J. Clinical impact and risk factors of intensive care unit-acquired nosocomial infection: a propensity score-matching study from 2018 to 2020 in a teaching Hospital in China. Infect Drug Resist. (2023) 16:569–79. doi: 10.2147/IDR.S394269, PMID: 36726386 PMC9885966

[3] Li RJ Wu YL Huang K Hu XQ Zhang JJ Yang LQ. A prospective surveillance study of healthcare-associated infections in an intensive care unit from a tertiary care teaching hospital from 2012-2019. Medicine. (2023) 102:e34469. doi: 10.1097/MD.0000000000034469 37543835 PMC10402966

[4] Gastmeier P Kola A Schwab F Behnke M Geffers C. Etiology of nosocomial infections in intensive care patients in German hospitals: an analysis of trends between 2008 and 2022. Int J Med Microbiol. (2024) 314:151594. doi: 10.1016/j.ijmm.2023.151594 38154413

[5] Wang Z Du M Cao H Yao H Liu B Bai Y. Epidemiology and risk factors of nosocomial infections in a Chinese tertiary-care hospital: a 10-year retrospective case-control study. Infect Dis. (2024) 56:320–9. doi: 10.1080/23744235.2024.2310647, PMID: 38317598

[6] Baddal B Taner F Ozsahin DU. Harnessing of artificial intelligence for the diagnosis and prevention of hospital-acquired infections: a systematic review. Diagnostics. (2024) 14:484. doi: 10.3390/diagnostics14050484, PMID: 38472956 PMC10930720

[7] Biesheuvel LA Dongelmans DA Elbers PWG. Artificial intelligence to advance acute and intensive care medicine. Curr Opin Crit Care. (2024) 30:246–50. doi: 10.1097/MCC.0000000000001150 38525882 PMC11064910

[8] Samadani A Wang T van Zon K Celi LA. VAP risk index: early prediction and hospital phenotyping of ventilator-associated pneumonia using machine learning. Artif Intel Med. (2023) 146:102715. doi: 10.1016/j.artmed.2023.102715 38042602

[9] Frondelius T Atkova I Miettunen J Rello J Vesty G Chew HSJ. Early prediction of ventilator-associated pneumonia with machine learning models: a systematic review and meta-analysis of prediction model performance. Eur J Intern Med. (2024) 121:76–87. doi: 10.1016/j.ejim.2023.11.009, PMID: 37981529

[10] Gao T Nong Z Luo Y Mo M Chen Z Yang Z. Machine learning-based prediction of in-hospital mortality for critically ill patients with sepsis-associated acute kidney injury. Ren Fail. (2024) 46:2316267. doi: 10.1080/0886022X.2024.2316267, PMID: 38369749 PMC10878338

[11] Barchitta M Maugeri A Favara G Riela PM Gallo G Mura I. A machine learning approach to predict healthcare-associated infections at intensive care unit admission: findings from the SPIN-UTI project. J Hosp Infect. (2021) 112:77–86. doi: 10.1016/j.jhin.2021.02.025, PMID: 33676936

[12] Barchitta M Maugeri A Favara G Riela PM Gallo G Mura I. Early prediction of seven-day mortality in intensive care unit using a machine learning model: results from the SPIN-UTI project. J Clin Med. (2021) 10:992. doi: 10.3390/jcm10050992, PMID: 33801207 PMC7957866

[13] Beltempo M Bresson G Lacroix G. Using machine learning to predict nosocomial infections and medical accidents in a NICU. Heal Technol. (2023) 13:75–87. doi: 10.1007/s12553-022-00723-1

[14] Ministry of Health of the People’s Republic of China. Healthcare-associated infections diagnosis criteria (trial). Nat Med J China. (2001) 5:61–7.

[15] Kumpf O Assenheimer M Bloos F Brauchle M Braun JP Brinkmann A. Quality indicators in intensive care medicine for Germany—fourth edition 2022. Ger Med Sci. (2023) 21:Doc10. doi: 10.3205/000324 37426886 PMC10326525

[16] Zhang Q Wang J Liu G Zhang W. Artificial intelligence can use physiological parameters to optimize treatment strategies and predict clinical deterioration of sepsis in ICU. Physiol Meas. (2023) 44:15003. doi: 10.1088/1361-6579/acb03b, PMID: 36599174

[17] National Health Commission of the People’s Republic of China. Collection and transportation of clinical microbiology test samples. Available at: http://www.nhc.gov.cn/wjw/s9492/201812/f1c15b1b58bc45729f8f9afc164b7805.shtml

[18] Zhang Q Chen G Zhu Q Liu Z Li Y Li R. Construct validation of machine learning for accurately predicting the risk of postoperative surgical site infection following spine surgery. J Hosp Infect. (2024) 146:232–41. doi: 10.1016/j.jhin.2023.09.024, PMID: 38029857

[19] Haskins IN Tamer R Phillips SE Thorson FC Kothari VM Perez AJ. Association of active smoking on 30-day wound events and additional morbidity and mortality following inguinal hernia repair with mesh: an analysis of the ACHQC database. Hernia. (2024) 28:33–42. doi: 10.1007/s10029-023-02886-w, PMID: 37776406

[20] Rafalko N Webster JL Jacob G Kutzler MA Goldstein ND. Generalizability of predictive models for Clostridioides difficile infection, severity and recurrence at an urban safety-net hospital. J Hosp Infect. (2024) 146:10–20. doi: 10.1016/j.jhin.2024.01.001 38219834

[21] Shrivastava T Singh V Agrawal A. Autism spectrum disorder detection with kNN imputer and machine learning classifiers via questionnaire mode of screening. Health Inform Sci Syst. (2024) 12:18. doi: 10.1007/s13755-024-00277-8, PMID: 38464462 PMC10917726

[22] Saeed MH Hama JI. Cardiac disease prediction using AI algorithms with SelectKBest. Med Biol Eng Comput. (2023) 61:3397–408. doi: 10.1007/s11517-023-02918-8 37679578

[23] Sun X. Supervised machine learning for exploratory analysis in family research. J Marriage Fam. (2024). doi: 10.1111/jomf.12973

[24] Chen T Guestrin C. XGBoost: a scalable tree boosting system. in: kdd’16: proceedings of the 22nd acm sigkdd international conference on knowledge discovery and data mining. assoc comp machinery; assoc comp machinery sigmod; Assoc Comp Machinery SIGKDD; (2016). p. 785–794.

[25] Zhang Y Xu J Zhang C Zhang X Yuan X Ni W. Community screening for dementia among older adults in China: a machine learning-based strategy. BMC Public Health. (2024) 24:1206. doi: 10.1186/s12889-024-18692-7 38693495 PMC11062005

[26] Ke G Meng Q Finley T Wang T Chen W Ma W. LightGBM: a highly efficient gradient boosting decision tree In: Guyon I Luxburg U Bengio S Wallach H Fergus R Vishwanathan S, editors. Proceedings of the 31st international conference on neural information processing systems (NIPS’17). Red Hook, NY, USA:Curran Associates Inc, (2017) 3149–3157.

[27] Musa F Prasad R. Predicting preeclampsia using principal component analysis and decision tree classifier. Curr Womens Health Rev. (2024) 20:14–15. doi: 10.2174/1573404820666230227120828

[28] Monteverde-Suarez D Gonzalez-Flores P Santos-Solorzano R Garcia-Minjares M Zavala-Sierra I de la Luz VL. Predicting students’ academic progress and related attributes in first-year medical students: an analysis with artificial neural networks and Naïve Bayes. BMC Med Educ. (2024) 24:74. doi: 10.1186/s12909-023-04918-6, PMID: 38243257 PMC10799512

[29] Prokhorenkova L Gusev G Vorobev A Dorogush AV Gulin A. CatBoost: unbiased boosting with categorical features In: Bengio S Wallach H Larochelle H Grauman K CesaBianchi N Garnett R, editors. Proceedings of the 32nd international conference on neural information processing systems (NIPS'18). Red Hook, NY, USA: Curran Associates Inc. (2018) 31:6639–6649.

[30] Mungan I Bektas S Cavus MA Sari S Turan S. The predictive power of SAPS-3 and SOFA scores and their relations with patient outcomes in the surgical intensive care unit. Turkish J Surg. (2019) 35:124–30. doi: 10.5578/turkjsurg.4223 PMC6796077 32550317

[31] Martos-Benitez FD Larrondo-Muguercia H Leon-Perez D Rivero-Lopez JC Orama-Requejo V Martinez-Alfonso JL. Performance of three prognostic models in critically ill patients with cancer: a prospective study. Int J Clin Oncol. (2020) 25:1242–9. doi: 10.1007/s10147-020-01659-0 32212014

[32] Hong N Liu C Gao J Han L Chang F Gong M. State of the art of machine learning–enabled clinical decision support in intensive care units: literature review. JMIR Med Inform. (2022) 10:e28781. doi: 10.2196/28781, PMID: 35238790 PMC8931648

[33] De Corte T Van Hoecke S De Waele J. Artificial intelligence in infection management in the ICU. Crit Care. (2022) 26:79. doi: 10.1186/s13054-022-03916-2 35337363 PMC8951654

[34] Ferrara M Bertozzi G Di Fazio N Aquila I Di Fazio A Maiese A. Risk management and patient safety in the artificial intelligence era: a systematic review. Healthcare. (2024) 12:549. doi: 10.3390/healthcare12050549 38470660 PMC10931321

[35] Zhu Y Simon GJ Wick EC Abe-Jones Y Najafi N Sheka A. Applying machine learning across sites: external validation of a surgical site infection detection algorithm. J Am Coll Surg. (2021) 232:963–971e1. e1. doi: 10.1016/j.jamcollsurg.2021.03.026, PMID: 33831539 PMC8679130

[36] European Centre for Disease Prevention and Control. Healthcare-associated infections. (Accessed May 22, 2024). Available at: https://www.ecdc.europa.eu/en/healthcare-associated-infections

[37] Stamm WE. Catheter-associated urinary tract infections: epidemiology, pathogenesis, and prevention. Am J Med. (1991) 91:65S–71S. doi: 10.1016/0002-9343(91)90345-x 1928194

[38] Al Nasser W El-Saed A Al-Jardani A Althaqafi A Alansari H Alsalman J. Rates of catheter-associated urinary tract infection in tertiary care hospitals in 3 Arabian gulf countries: a 6-year surveillance study. Am J Infect Control. (2016) 44:1589–94. doi: 10.1016/j.ajic.2016.06.030 27692786

[39] Cabrera-Tejada GG Chico-Sánchez P Gras-Valentí P Jaime-Sánchez FA Galiana-Ivars M Balboa-Esteve S. Estimation of additional costs in patients with ventilator-associated pneumonia. Antibiotics. (2024) 13:2. doi: 10.3390/antibiotics13010002 PMC10812792 38275312

[40] Karajizadeh M Nasiri M Yadollahi M Zolfaghari AH Pakdam A. Mortality prediction from hospital-acquired infections in trauma patients using an unbalanced dataset. Healthc Inform Res. (2020) 26:284–94. doi: 10.4258/hir.2020.26.4.284 33190462 PMC7674815

[41] Krawczyk B. Learning from imbalanced data: open challenges and future directions. Prog Artif Intell. (2016) 5:221–32. doi: 10.1007/s13748-016-0094-0

[42] Cho SY Kim Z Chung DR Cho BH Chung MJ Kim JH. Development of machine learning models for the surveillance of colon surgical site infections. J Hosp Infect. (2024) 146:224–31. doi: 10.1016/j.jhin.2023.03.025, PMID: 37094715

[43] National Health Commission of the People’s Republic of China. Intensive Care Unit Hospital Infection Prevention and Control Guidelines. Available at: http://www.nhc.gov.cn/wjw/s9496/201701/1f9de66563304061a4fcd7f54a9399fb.shtml

[44] Ding Y Ji Z Liu Y Niu J. Braden scale for predicting pneumonia after spontaneous intracerebral hemorrhage. Rev Assoc Med Bras. (2022) 68:904–11. doi: 10.1590/1806-9282.20211339 35946766 PMC9574960

[45] Jin X Wu S Bai Y. Risk factors and characteristics of bacterial infection during first-line chemotherapy for metastatic gastric or gastroesophageal junction adenocarcinoma. Support Care Cancer. (2022) 30:2121–9. doi: 10.1007/s00520-021-06557-3 34677650

[46] Arzilli G De Vita E Pasquale M Carloni LM Pellegrini M Di Giacomo M. Innovative techniques for infection control and surveillance in hospital settings and long-term care facilities: a scoping review. Antibiotics. (2024) 13:77. doi: 10.3390/antibiotics13010077 38247635 PMC10812752

[47] Feng T Noren DP Kulkarni C Mariani S Zhao C Ghosh E. Machine learning-based clinical decision support for infection risk prediction. Front Med. (2023) 10:1213411. doi: 10.3389/fmed.2023.1213411 PMC10765581 38179280

