# Supplementary material for: Development and evaluation of a model for predicting the risk of healthcare-associated infections in patients admitted to intensive care units
Source: Front Public Health. 2024 Sep 12;12:1444176. doi: 10.3389/fpubh.2024.1444176 (PMC11424534; doi:10.3389/fpubh.2024.1444176)
Supplement: Supplementary file 2 [file Table_2.docx]

{

"cells": [

{

"cell_type": "code",

"execution_count": 1,

"id": "ca9391d7-b458-463d-b9ca-b4a182ccfac6",

"metadata": {},

"outputs": [

{

"name": "stderr",

"output_type": "stream",

"text": [

"\n",

"KeyboardInterrupt\n",

"\n"

]

}

],

"source": [

"# The receiver-operating characteristic curves for ten ML models without undergoing SMOTE oversampling processing\n",

"import pandas as pd\n",

"from sklearn.preprocessing import OneHotEncoder, StandardScaler\n",

"from sklearn.compose import ColumnTransformer\n",

"from imblearn.over_sampling import SMOTE\n",

"from sklearn.model_selection import train_test_split\n",

"from sklearn.ensemble import RandomForestClassifier, AdaBoostClassifier, GradientBoostingClassifier\n",

"from sklearn.tree import DecisionTreeClassifier\n",

"from sklearn.linear_model import LogisticRegression\n",

"from sklearn.naive_bayes import GaussianNB\n",

"from sklearn.neural_network import MLPClassifier\n",

"from sklearn.metrics import roc_curve, roc_auc_score\n",

"import matplotlib.pyplot as plt\n",

"import lightgbm as lgb\n",

"import xgboost as xgb\n",

"from catboost import CatBoostClassifier\n",

"from sklearn.feature_selection import SelectFromModel\n",

"from sklearn.pipeline import Pipeline\n",

"from sklearn.model_selection import cross_val_score\n",

"from sklearn.feature_selection import SelectFromModel\n",

"from sklearn.pipeline import Pipeline\n",

"from sklearn.feature_selection import SelectFromModel\n",

"from sklearn.pipeline import Pipeline\n",

"from sklearn.feature_selection import SelectFromModel\n",

"from sklearn.pipeline import Pipeline\n",

"from sklearn.feature_selection import SelectFromModel\n",

"from sklearn.pipeline import Pipeline\n",

"\n",

"\n",

"file_path = r'D:\\2024_4_29.xlsx'\n",

"data = pd.read_excel(file_path)\n",

"\n",

"\n",

"features = ['性别', '糖尿病', '年龄', '白细胞计数结果标志', '单核细胞计数结果标志', '红细胞结果标志', \n",

" '淋巴细胞计数结果标志', '血红蛋白结果标志', '血小板计数结果标志', '葡萄糖结果标志', \n",

" '白蛋白结果标志', '总胆红素结果标志', \"C反应蛋白结果标志\", \"RBC体积分布宽度结果标志\", \n",

" \"红细胞压积结果标志\", \"平均RBC血红蛋白含量结果标志\", \"平均RBC血红蛋白浓度结果标志\", \n",

" \"平均红细胞体积结果标志\", 'Braden评分（压疮评分）', 'NRS2002评分（营养风险筛查）', \n",

" 'GlasGow评分（昏迷评分）', 'CPOT评分（疼痛评分表）', '误吸/窒息评分', '肠内营养耐受性', \n",

" 'VTE评分（静脉血栓栓塞症风险）', '非计划拔管评估']\n",

"target = '院感'\n",

"\n",

"X = data[features]\n",

"y = data[target]\n",

"\n",

"\n",

"categorical_features = ['白细胞计数结果标志', '单核细胞计数结果标志', '红细胞结果标志', \n",

" '淋巴细胞计数结果标志', '血红蛋白结果标志', '血小板计数结果标志', '葡萄糖结果标志', \n",

" '白蛋白结果标志', '总胆红素结果标志', \"C反应蛋白结果标志\", \"RBC体积分布宽度结果标志\", \n",

" \"红细胞压积结果标志\", \"平均RBC血红蛋白含量结果标志\", \"平均RBC血红蛋白浓度结果标志\", \n",

" \"平均红细胞体积结果标志\"]\n",

"categorical_transformer = OneHotEncoder()\n",

"\n",

"\n",

"numeric_features = ['年龄', 'Braden评分（压疮评分）', 'NRS2002评分（营养风险筛查）', 'GlasGow评分（昏迷评分）',\n",

" 'CPOT评分（疼痛评分表）', '误吸/窒息评分', '肠内营养耐受性', 'VTE评分（静脉血栓栓塞症风险）', '非计划拔管评估']\n",

"numeric_transformer = StandardScaler()\n",

"\n",

"preprocessor = ColumnTransformer(\n",

" transformers=[\n",

" ('num', numeric_transformer, numeric_features),\n",

" ('cat', categorical_transformer, categorical_features)\n",

" ])\n",

"\n",

"\n",

"pipeline = Pipeline(steps=[('preprocessor', preprocessor),\n",

" ('feature_selection', SelectFromModel(LogisticRegression(penalty=\"l1\", solver='liblinear')))])\n",

"\n",

"\n",

"X_selected = pipeline.fit_transform(X, y)\n",

"\n",

"\n",

"X_train, X_test, y_train, y_test = train_test_split(X_selected, y, test_size=0.2, random_state=42)\n",

"\n",

"\n",

"classifiers = {\n",

" \"Random Forest\": RandomForestClassifier(),\n",

" \"XGBoost\": xgb.XGBClassifier(),\n",

" \"MLP\": MLPClassifier(),\n",

" \"LightGBM\": lgb.LGBMClassifier(),\n",

" \"Logistic Regression\": LogisticRegression(),\n",

" \"Decision tree\": DecisionTreeClassifier(),\n",

" \"Naive bayes\": GaussianNB(),\n",

" \"AdaBoost\": AdaBoostClassifier(),\n",

" \"CatBoost\": CatBoostClassifier(verbose=False),\n",

" \"Neural Networks\": MLPClassifier()\n",

"}\n",

"\n",

"\n",

"results = {}\n",

"for name, clf in classifiers.items():\n",

" clf.fit(X_train, y_train)\n",

" y_pred = clf.predict_proba(X_test)[:, 1]\n",

" fpr, tpr, thresholds = roc_curve(y_test, y_pred)\n",

" auc = roc_auc_score(y_test, y_pred)\n",

" results[name] = {\"fpr\": fpr, \"tpr\": tpr, \"auc\": auc}\n",

"\n",

"\n",

"plt.figure(figsize=(10, 8))\n",

"\n",

"sorted_results = sorted(results.items(), key=lambda x: x[1][\"auc\"], reverse=True)\n",

"for name, result in sorted_results:\n",

" plt.plot(result[\"fpr\"], result[\"tpr\"], label=f'{name} (AUC = {result[\"auc\"]:.3f})')\n",

"plt.plot([0, 1], [0, 1], linestyle='--', color='gray', label='Random Guessing')\n",

"plt.xlabel('False Positive Rate')\n",

"plt.ylabel('True Positive Rate')\n",

"plt.title('Receiver Operating Characteristic (ROC) Curve')\n",

"plt.legend(loc='lower right')\n",

"plt.grid(True)\n",

"plt.show()\n"

]

},

{

"cell_type": "code",

"execution_count": null,

"id": "2dc82626-dc96-4916-9187-6ce9f0caa38b",

"metadata": {},

"outputs": [],

"source": [

"# The ten-fold cross-validation results of the CatBoost model without SMOTE oversampling.\n",

"from sklearn.model_selection import cross_val_predict\n",

"from sklearn.model_selection import StratifiedKFold\n",

"from sklearn import metrics\n",

"import numpy as np\n",

"\n",

"\n",

"clf = CatBoostClassifier(verbose=False)\n",

"\n",

"\n",

"cv = StratifiedKFold(n_splits=10, shuffle=True, random_state=42)\n",

"\n",

"\n",

"fold_results = []\n",

"\n",

"\n",

"for i, (train_index, test_index) in enumerate(cv.split(X_selected, y)):\n",

" X_train, X_test = X_selected[train_index], X_selected[test_index]\n",

" y_train, y_test = y[train_index], y[test_index]\n",

" \n",

" clf.fit(X_train, y_train)\n",

" y_prob = clf.predict_proba(X_test)[:, 1]\n",

" fpr, tpr, _ = metrics.roc_curve(y_test, y_prob)\n",

" roc_auc = metrics.auc(fpr, tpr)\n",

" fold_results.append({\"fpr\": fpr, \"tpr\": tpr, \"auc\": roc_auc, \"fold\": i+1})\n",

"\n",

"tprs = []\n",

"aucs = []\n",

"mean_fpr = np.linspace(0, 1, 100)\n",

"plt.figure(figsize=(10, 8))\n",

"\n",

"for result in fold_results:\n",

" plt.plot(result[\"fpr\"], result[\"tpr\"], alpha=0.3, label=f'Fold {result[\"fold\"]} (AUC = {result[\"auc\"]:.3f})')\n",

" interp_tpr = np.interp(mean_fpr, result[\"fpr\"], result[\"tpr\"])\n",

" interp_tpr[0] = 0.0\n",

" tprs.append(interp_tpr)\n",

" aucs.append(result[\"auc\"])\n",

"\n",

"mean_tpr = np.mean(tprs, axis=0)\n",

"mean_tpr[-1] = 1.0\n",

"mean_auc = metrics.auc(mean_fpr, mean_tpr)\n",

"\n",

"std_auc = np.std(aucs)\n",

"\n",

"tpr_upper = np.minimum(mean_tpr + 1.96 * np.std(tprs, axis=0), 1)\n",

"tpr_lower = np.maximum(mean_tpr - 1.96 * np.std(tprs, axis=0), 0)\n",

"\n",

"plt.plot(mean_fpr, mean_tpr, color='b', label=f'Mean ROC (AUC = {mean_auc:.3f} $\\pm$ {std_auc:.3f})')\n",

"plt.fill_between(mean_fpr, tpr_lower, tpr_upper, color='grey', alpha=0.3)\n",

"\n",

"plt.plot([0, 1], [0, 1], linestyle='--', color='gray', label='Random Guessing')\n",

"plt.xlabel('False Positive Rate')\n",

"plt.ylabel('True Positive Rate')\n",

"plt.title('Receiver Operating Characteristic (ROC) Curve')\n",

"plt.legend(loc='lower right')\n",

"plt.grid(True)\n",

"\n",

"plt.show()"

]

},

{

"cell_type": "code",

"execution_count": null,

"id": "311066ad-8866-46b4-bab9-df0b2a1922d7",

"metadata": {},

"outputs": [],

"source": [

"# The receiver-operating characteristic curves for ten ML models with undergoing SMOTE oversampling processing\n",

"import pandas as pd\n",

"from sklearn.preprocessing import OneHotEncoder, StandardScaler\n",

"from sklearn.compose import ColumnTransformer\n",

"from imblearn.over_sampling import SMOTE\n",

"from sklearn.model_selection import train_test_split\n",

"from sklearn.ensemble import RandomForestClassifier, AdaBoostClassifier, GradientBoostingClassifier\n",

"from sklearn.tree import DecisionTreeClassifier\n",

"from sklearn.linear_model import LogisticRegression\n",

"from sklearn.naive_bayes import GaussianNB\n",

"from sklearn.neural_network import MLPClassifier\n",

"from sklearn.metrics import roc_curve, roc_auc_score\n",

"import matplotlib.pyplot as plt\n",

"import lightgbm as lgb\n",

"import xgboost as xgb\n",

"from catboost import CatBoostClassifier\n",

"from sklearn.feature_selection import SelectFromModel\n",

"from sklearn.pipeline import Pipeline\n",

"\n",

"\n",

"file_path = r'D:\\2024_4_29.xlsx'\n",

"data = pd.read_excel(file_path)\n",

"\n",

"\n",

"features = ['性别', '糖尿病', '年龄', '白细胞计数结果标志', '单核细胞计数结果标志', '红细胞结果标志', \n",

" '淋巴细胞计数结果标志', '血红蛋白结果标志', '血小板计数结果标志', '葡萄糖结果标志', \n",

" '白蛋白结果标志', '总胆红素结果标志', \"C反应蛋白结果标志\", \"RBC体积分布宽度结果标志\", \n",

" \"红细胞压积结果标志\", \"平均RBC血红蛋白含量结果标志\", \"平均RBC血红蛋白浓度结果标志\", \n",

" \"平均红细胞体积结果标志\", 'Braden评分（压疮评分）', 'NRS2002评分（营养风险筛查）', \n",

" 'GlasGow评分（昏迷评分）', 'CPOT评分（疼痛评分表）', '误吸/窒息评分', '肠内营养耐受性', \n",

" 'VTE评分（静脉血栓栓塞症风险）', '非计划拔管评估']\n",

"target = '院感'\n",

"\n",

"X = data[features]\n",

"y = data[target]\n",

"\n",

"\n",

"categorical_features = ['白细胞计数结果标志', '单核细胞计数结果标志', '红细胞结果标志', \n",

" '淋巴细胞计数结果标志', '血红蛋白结果标志', '血小板计数结果标志', '葡萄糖结果标志', \n",

" '白蛋白结果标志', '总胆红素结果标志', \"C反应蛋白结果标志\", \"RBC体积分布宽度结果标志\", \n",

" \"红细胞压积结果标志\", \"平均RBC血红蛋白含量结果标志\", \"平均RBC血红蛋白浓度结果标志\", \n",

" \"平均红细胞体积结果标志\"]\n",

"categorical_transformer = OneHotEncoder()\n",

"\n",

"\n",

"numeric_features = ['年龄', 'Braden评分（压疮评分）', 'NRS2002评分（营养风险筛查）', 'GlasGow评分（昏迷评分）',\n",

" 'CPOT评分（疼痛评分表）', '误吸/窒息评分', '肠内营养耐受性', 'VTE评分（静脉血栓栓塞症风险）', '非计划拔管评估']\n",

"\n",

"numeric_transformer = StandardScaler()\n",

"\n",

"\n",

"preprocessor = ColumnTransformer(\n",

" transformers=[\n",

" ('num', numeric_transformer, numeric_features),\n",

" ('cat', categorical_transformer, categorical_features)\n",

" ])\n",

"\n",

"\n",

"X_preprocessed = preprocessor.fit_transform(X)\n",

"\n",

"\n",

"smote = SMOTE(random_state=42)\n",

"X_resampled, y_resampled = smote.fit_resample(X_preprocessed, y)\n",

"\n",

"\n",

"selector = SelectFromModel(LogisticRegression(penalty=\"l1\", solver='liblinear'))\n",

"X_selected = selector.fit_transform(X_resampled, y_resampled)\n",

"\n",

"\n",

"X_train, X_test, y_train, y_test = train_test_split(X_selected, y_resampled, test_size=0.2, random_state=42)\n",

"\n",

"\n",

"classifiers = {\n",

" \"Random Forest\": RandomForestClassifier(),\n",

" \"XGBoost\": xgb.XGBClassifier(),\n",

" \"MLP\": MLPClassifier(),\n",

" \"LightGBM\": lgb.LGBMClassifier(),\n",

" \"Logistic Regression\": LogisticRegression(),\n",

" \"Decision tree\": DecisionTreeClassifier(),\n",

" \"Naive bayes\": GaussianNB(),\n",

" \"AdaBoost\": AdaBoostClassifier(),\n",

" \"CatBoost\": CatBoostClassifier(verbose=False),\n",

" \"Neural Networks\": MLPClassifier()\n",

"}\n",

"\n",

"\n",

"results = {}\n",

"for name, clf in classifiers.items():\n",

" clf.fit(X_train, y_train)\n",

" y_pred = clf.predict_proba(X_test)[:, 1]\n",

" fpr, tpr, thresholds = roc_curve(y_test, y_pred)\n",

" auc = roc_auc_score(y_test, y_pred)\n",

" results[name] = {\"fpr\": fpr, \"tpr\": tpr, \"auc\": auc}\n",

"\n",

"\n",

"sorted_results = sorted(results.items(), key=lambda x: x[1][\"auc\"], reverse=True)\n",

"\n",

"\n",

"plt.figure(figsize=(10, 8))\n",

"for name, result in sorted_results:\n",

" plt.plot(result[\"fpr\"], result[\"tpr\"], label=f'{name} (AUC = {result[\"auc\"]:.3f})')\n",

"plt.plot([0, 1], [0, 1], linestyle='--', color='gray', label='Random Guessing')\n",

"plt.xlabel('False Positive Rate')\n",

"plt.ylabel('True Positive Rate')\n",

"plt.title('Receiver Operating Characteristic (ROC) Curve')\n",

"plt.legend(loc='lower right')\n",

"plt.grid(True)\n",

"plt.show()\n"

]

},

{

"cell_type": "code",

"execution_count": null,

"id": "e1679ce0-2224-47fe-b52f-ae55b13f1976",

"metadata": {},

"outputs": [],

"source": [

"# The ten-fold cross-validation results of the CatBoost model with SMOTE oversampling\n",

"import pandas as pd\n",

"from sklearn.preprocessing import OneHotEncoder, StandardScaler\n",

"from sklearn.compose import ColumnTransformer\n",

"from imblearn.over_sampling import SMOTE\n",

"from sklearn.model_selection import train_test_split\n",

"from sklearn.ensemble import RandomForestClassifier, AdaBoostClassifier, GradientBoostingClassifier\n",

"from sklearn.tree import DecisionTreeClassifier\n",

"from sklearn.linear_model import LogisticRegression\n",

"from sklearn.naive_bayes import GaussianNB\n",

"from sklearn.neural_network import MLPClassifier\n",

"from sklearn.metrics import roc_curve, roc_auc_score\n",

"import matplotlib.pyplot as plt\n",

"import lightgbm as lgb\n",

"import xgboost as xgb\n",

"from catboost import CatBoostClassifier\n",

"from sklearn.feature_selection import SelectFromModel\n",

"from sklearn.pipeline import Pipeline\n",

"\n",

"\n",

"file_path = r'D:\\2024_4_29.xlsx'\n",

"data = pd.read_excel(file_path)\n",

"\n",

"features = ['性别', '糖尿病', '年龄', '白细胞计数结果标志', '单核细胞计数结果标志', '红细胞结果标志', \n",

" '淋巴细胞计数结果标志', '血红蛋白结果标志', '血小板计数结果标志', '葡萄糖结果标志', \n",

" '白蛋白结果标志', '总胆红素结果标志', \"C反应蛋白结果标志\", \"RBC体积分布宽度结果标志\", \n",

" \"红细胞压积结果标志\", \"平均RBC血红蛋白含量结果标志\", \"平均RBC血红蛋白浓度结果标志\", \n",

" \"平均红细胞体积结果标志\", 'Braden评分（压疮评分）', 'NRS2002评分（营养风险筛查）', \n",

" 'GlasGow评分（昏迷评分）', 'CPOT评分（疼痛评分表）', '误吸/窒息评分', '肠内营养耐受性', \n",

" 'VTE评分（静脉血栓栓塞症风险）', '非计划拔管评估',\"嗜碱性粒细胞计数结果标志\",\n",

" \"嗜酸性粒细胞计数结果标志\",\"血小板分布宽度结果标志\",\"血小板压积结果标志\",\"中性粒细胞计数结果标志\"]\n",

"target = '院感'\n",

"\n",

"X = data[features]\n",

"y = data[target]\n",

"\n",

"\n",

"categorical_features = ['白细胞计数结果标志', '单核细胞计数结果标志', '红细胞结果标志', \n",

" '淋巴细胞计数结果标志', '血红蛋白结果标志', '血小板计数结果标志', '葡萄糖结果标志', \n",

" '白蛋白结果标志', '总胆红素结果标志', \"C反应蛋白结果标志\", \"RBC体积分布宽度结果标志\", \n",

" \"红细胞压积结果标志\", \"平均RBC血红蛋白含量结果标志\", \"平均RBC血红蛋白浓度结果标志\", \n",

" \"平均红细胞体积结果标志\",\"嗜碱性粒细胞计数结果标志\",\"嗜酸性粒细胞计数结果标志\",\n",

" \"血小板分布宽度结果标志\",\"血小板压积结果标志\",\"中性粒细胞计数结果标志\"]\n",

"categorical_transformer = OneHotEncoder()\n",

"\n",

"\n",

"numeric_features = ['年龄', 'Braden评分（压疮评分）', 'NRS2002评分（营养风险筛查）', 'GlasGow评分（昏迷评分）',\n",

" 'CPOT评分（疼痛评分表）', '误吸/窒息评分', '肠内营养耐受性', 'VTE评分（静脉血栓栓塞症风险）', '非计划拔管评估']\n",

"numeric_transformer = StandardScaler()\n",

"\n",

"\n",

"preprocessor = ColumnTransformer(\n",

" transformers=[\n",

" ('num', numeric_transformer, numeric_features),\n",

" ('cat', categorical_transformer, categorical_features)\n",

" ])\n",

"\n",

"\n",

"X_preprocessed = preprocessor.fit_transform(X)\n",

"\n",

"\n",

"smote = SMOTE(random_state=42)\n",

"X_resampled, y_resampled = smote.fit_resample(X_preprocessed, y)\n",

"\n",

"selector = SelectFromModel(LogisticRegression(penalty=\"l1\", solver='liblinear'))\n",

"X_selected = selector.fit_transform(X_resampled, y_resampled)\n",

"\n",

"\n",

"X_train, X_test, y_train, y_test = train_test_split(X_selected, y_resampled, test_size=0.2, random_state=42)\n",

"\n",

"\n",

"classifiers = {\n",

" \"Random Forest\": RandomForestClassifier(),\n",

" \"XGBoost\": xgb.XGBClassifier(),\n",

" \"MLP\": MLPClassifier(),\n",

" \"LightGBM\": lgb.LGBMClassifier(),\n",

" \"Logistic Regression\": LogisticRegression(),\n",

" \"Decision tree\": DecisionTreeClassifier(),\n",

" \"Naive bayes\": GaussianNB(),\n",

" \"AdaBoost\": AdaBoostClassifier(),\n",

" \"CatBoost\": CatBoostClassifier(verbose=False),\n",

" \"Neural Networks\": MLPClassifier()\n",

"}\n",

"\n",

"\n",

"results = {}\n",

"for name, clf in classifiers.items():\n",

" clf.fit(X_train, y_train)\n",

" y_pred = clf.predict_proba(X_test)[:, 1]\n",

" fpr, tpr, thresholds = roc_curve(y_test, y_pred)\n",

" auc = roc_auc_score(y_test, y_pred)\n",

" results[name] = {\"fpr\": fpr, \"tpr\": tpr, \"auc\": auc}\n",

"\n",

"\n",

"sorted_results = sorted(results.items(), key=lambda x: x[1][\"auc\"], reverse=True)\n",

"\n",

"\n",

"plt.figure(figsize=(10, 8))\n",

"catboost_result = results[\"CatBoost\"]\n",

"plt.plot(catboost_result[\"fpr\"], catboost_result[\"tpr\"], label=f'CatBoost (AUC = {catboost_result[\"auc\"]:.3f})')\n",

"plt.plot([0, 1], [0, 1], linestyle='--', color='gray', label='Random Guessing')\n",

"plt.xlabel('False Positive Rate')\n",

"plt.ylabel('True Positive Rate')\n",

"plt.title('Receiver Operating Characteristic (ROC) Curve for CatBoost')\n",

"plt.legend(loc='lower right')\n",

"plt.grid(True)\n",

"plt.show()"

]

},

{

"cell_type": "code",

"execution_count": null,

"id": "6161c23c-1c36-4d2b-820a-6f7929e6f428",

"metadata": {},

"outputs": [],

"source": []

},

{

"cell_type": "code",

"execution_count": null,

"id": "752e30be-d5a0-4a0e-903a-83f68944c870",

"metadata": {},

"outputs": [],

"source": [

"# SHapley Additive exPlanations\n",

"import pandas as pd\n",

"from imblearn.over_sampling import SMOTE\n",

"from sklearn.model_selection import train_test_split\n",

"from sklearn.ensemble import RandomForestClassifier, AdaBoostClassifier, GradientBoostingClassifier\n",

"from sklearn.tree import DecisionTreeClassifier\n",

"from sklearn.linear_model import LogisticRegression\n",

"from sklearn.naive_bayes import GaussianNB\n",

"from sklearn.neural_network import MLPClassifier\n",

"from sklearn.metrics import roc_curve, roc_auc_score\n",

"import matplotlib.pyplot as plt\n",

"import lightgbm as lgb\n",

"import xgboost as xgb\n",

"from catboost import CatBoostClassifier\n",

"from sklearn.feature_selection import SelectFromModel\n",

"\n",

"\n",

"file_path = r'D:\\2024_5_23.xlsx'\n",

"data = pd.read_excel(file_path)\n",

"\n",

"\n",

"features = ['Age','Braden score','NRS 2002 score','GCS score','PAS','Enteral feeding tolerance',\n",

"'CRS','Female','Male','High WBC count','Low WBC count','Medium WBC count',\n",

"'High RBC','Low RBC','Medium RBC','High lymphocyte count','Low lymphocyte count','Medium lymphocyte count',\n",

"'High hemoglobin level','Low hemoglobin level','Medium hemoglobin level','High total bilirubin level','Low total bilirubin level',\n",

"'Medium total bilirubin level','High RBC distribution width','Low RBC distribution width','Medium RBC distribution width','High hematocrit',\n",

"'Low hematocrit','Medium hematocrit','High mean corpuscular hemoglobin concentration','Low mean corpuscular hemoglobin concentration',\n",

"'Medium mean corpuscular hemoglobin concentration','High mean corpuscular volume','Low mean corpuscular volume','Medium mean corpuscular volume',\n",

"'High eosinophil count','low eosinophil count','Medium eosinophil count']\n",

"\n",

"target = 'HAI'\n",

"\n",

"X = data[features]\n",

"y = data[target]\n",

"\n",

"\n",

"smote = SMOTE(random_state=42)\n",

"X_resampled, y_resampled = smote.fit_resample(X, y)\n",

"\n",

"\n",

"selector = SelectFromModel(LogisticRegression(penalty=\"l1\", solver='liblinear'))\n",

"X_selected = selector.fit_transform(X_resampled, y_resampled)\n",

"\n",

"\n",

"X_train, X_test, y_train, y_test = train_test_split(X_selected, y_resampled, test_size=0.2, random_state=42)\n",

"\n",

"\n",

"classifiers = {\n",

" \"Random Forest\": RandomForestClassifier(),\n",

" \"XGBoost\": xgb.XGBClassifier(),\n",

" \"MLP\": MLPClassifier(),\n",

" \"LightGBM\": lgb.LGBMClassifier(),\n",

" \"Logistic Regression\": LogisticRegression(),\n",

" \"Decision tree\": DecisionTreeClassifier(),\n",

" \"Naive bayes\": GaussianNB(),\n",

" \"AdaBoost\": AdaBoostClassifier(),\n",

" \"CatBoost\": CatBoostClassifier(verbose=False),\n",

" \"Neural Networks\": MLPClassifier()\n",

"}\n",

"\n",

"\n",

"results = {}\n",

"for name, clf in classifiers.items():\n",

" clf.fit(X_train, y_train)\n",

" y_pred = clf.predict_proba(X_test)[:, 1]\n",

" fpr, tpr, thresholds = roc_curve(y_test, y_pred)\n",

" auc = roc_auc_score(y_test, y_pred)\n",

" results[name] = {\"fpr\": fpr, \"tpr\": tpr, \"auc\": auc}\n",

"\n",

"\n",

"sorted_results = sorted(results.items(), key=lambda x: x[1][\"auc\"], reverse=True)\n",

"\n",

"\n",

"plt.figure(figsize=(10, 8))\n",

"for name, result in sorted_results:\n",

" plt.plot(result[\"fpr\"], result[\"tpr\"], label=f'{name} (AUC = {result[\"auc\"]:.3f})')\n",

"plt.plot([0, 1], [0, 1], linestyle='--', color='gray', label='Random Guessing')\n",

"plt.xlabel('False Positive Rate')\n",

"plt.ylabel('True Positive Rate')\n",

"plt.title('Receiver Operating Characteristic (ROC) Curve')\n",

"plt.legend(loc='lower right')\n",

"plt.grid(True)\n",

"plt.show()\n",

"\n",

"import shap\n",

"import matplotlib.pyplot as plt\n",

"\n",

"\n",

"feature_names = features\n",

"\n",

"\n",

"explainer = shap.TreeExplainer(classifiers['CatBoost']) \n",

"shap_values = explainer.shap_values(X_test)\n",

"\n",

"\n",

"shap.summary_plot(shap_values, X_test, feature_names=feature_names, plot_type='violin', show=False)\n",

"plt.gcf().set_size_inches(20, 8) \n",

"\n",

"plt.show()"

]

},

{

"cell_type": "code",

"execution_count": null,

"id": "09e10c54-e8c0-426e-9031-9f63831cb5dc",

"metadata": {},

"outputs": [],

"source": []

}

],

"metadata": {

"kernelspec": {

"display_name": "Python 3 (ipykernel)",

"language": "python",

"name": "python3"

},

"language_info": {

"codemirror_mode": {

"name": "ipython",

"version": 3

},

"file_extension": ".py",

"mimetype": "text/x-python",

"name": "python",

"nbconvert_exporter": "python",

"pygments_lexer": "ipython3",

"version": "3.8.10"

}

},

"nbformat": 4,

"nbformat_minor": 5

"cells": [

{

"cell_type": "code",

"execution_count": null,

"id": "b3ba9dab-c635-4378-9f3f-b67850c1b733",

"metadata": {},

"outputs": [],

"source": [

"from sklearn.metrics import roc_curve, roc_auc_score, confusion_matrix\n",

"import numpy as np\n",

"\n",

"\n",

"catboost_clf = CatBoostClassifier(verbose=False)\n",

"catboost_clf.fit(X_train, y_train)\n",

"\n",

"\n",

"y_pred_proba = catboost_clf.predict_proba(X_test)[:, 1]\n",

"\n",

"\n",

"fpr, tpr, thresholds = roc_curve(y_test, y_pred_proba)\n",

"\n",

"\n",

"youden_j = tpr - fpr\n",

"optimal_idx = np.argmax(youden_j)\n",

"optimal_threshold = thresholds[optimal_idx]\n",

"\n",

"\n",

"print(f\"Optimal threshold for CatBoost model: {optimal_threshold}\")\n",

"\n",

"\n",

"y_pred_optimal = (y_pred_proba >= optimal_threshold).astype(int)\n",

"cm = confusion_matrix(y_test, y_pred_optimal)\n",

"print(\"Confusion Matrix at optimal threshold:\")\n",

"print(cm)"

]

}

],

"metadata": {

"kernelspec": {

"display_name": "Python 3 (ipykernel)",

"language": "python",

"name": "python3"

},

"language_info": {

"codemirror_mode": {

"name": "ipython",

"version": 3

},

"file_extension": ".py",

"mimetype": "text/x-python",

"name": "python",

"nbconvert_exporter": "python",

"pygments_lexer": "ipython3",

"version": "3.8.10"

}

},

"nbformat": 4,

"nbformat_minor": 5

}
