# Supplementary figures and images for: Development and evaluation of a model for predicting the risk of healthcare-associated infections in patients admitted to intensive care units
Source: Front Public Health. 2024 Sep 12;12:1444176. doi: 10.3389/fpubh.2024.1444176 (PMC11424534; doi:10.3389/fpubh.2024.1444176)

**Flowchart of the inclusion and exclusion criteria for the study population**


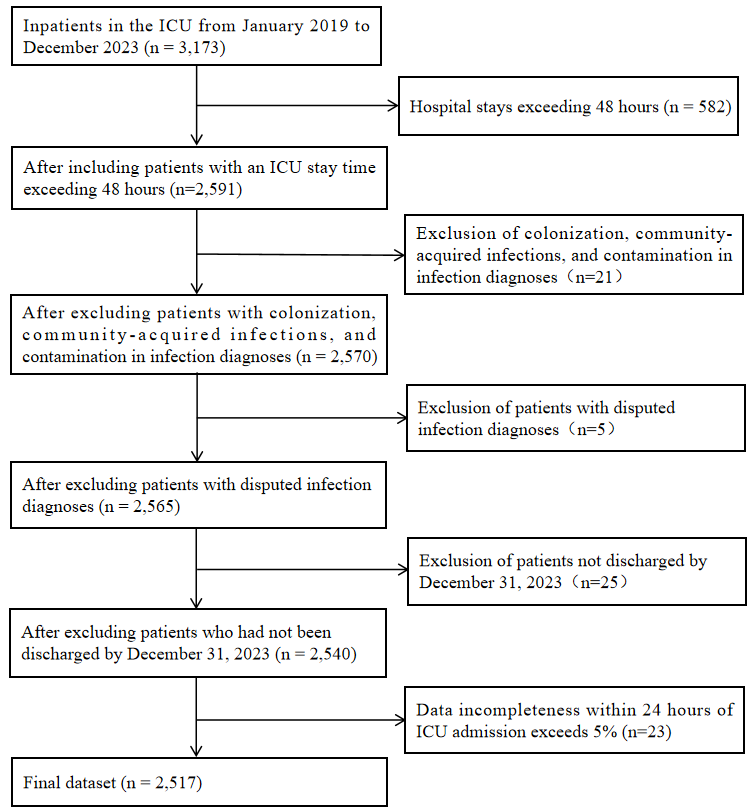

Supplement: Supplementary file 3 [file Data_Sheet_1.docx]
